# Update of Recommendations for Use of Once-Weekly Isoniazid-Rifapentine Regimen to Treat Latent *Mycobacterium tuberculosis* Infection

**DOI:** 10.15585/mmwr.mm6725a5

**Published:** 2018-06-29

**Authors:** Andrey S. Borisov, Sapna Bamrah Morris, Gibril J. Njie, Carla A. Winston, Deron Burton, Stefan Goldberg, Rachel Yelk Woodruff, Leeanna Allen, Philip LoBue, Andrew Vernon

**Affiliations:** 1 Division of Tuberculosis Elimination, National Center for HIV/AIDS, Viral Hepatitis, STD, and TB Prevention, CDC.

Treatment of latent tuberculosis infection (LTBI) is critical to the control and elimination of tuberculosis disease (TB) in the United States. In 2011, CDC recommended a short-course combination regimen of once-weekly isoniazid and rifapentine for 12 weeks (3HP) by directly observed therapy (DOT) for treatment of LTBI, with limitations for use in children aged <12 years and persons with human immunodeficiency virus (HIV) infection (*1*). CDC identified the use of 3HP in those populations, as well as self-administration of the 3HP regimen, as areas to address in updated recommendations. In 2017, a CDC Work Group conducted a systematic review and meta-analyses of the 3HP regimen using methods adapted from the Guide to Community Preventive Services. In total, 19 articles representing 15 unique studies were included in the meta-analysis, which determined that 3HP is as safe and effective as other recommended LTBI regimens and achieves substantially higher treatment completion rates. In July 2017, the Work Group presented the meta-analysis findings to a group of TB experts, and in December 2017, CDC solicited input from the Advisory Council for the Elimination of Tuberculosis (ACET) and members of the public for incorporation into the final recommendations. CDC continues to recommend 3HP for treatment of LTBI in adults and now recommends use of 3HP 1) in persons with LTBI aged 2–17 years; 2) in persons with LTBI who have HIV infection, including acquired immunodeficiency syndrome (AIDS), and are taking antiretroviral medications with acceptable drug-drug interactions with rifapentine; and 3) by DOT or self-administered therapy (SAT) in persons aged ≥2 years.

## Systematic Review

A CDC Work Group including epidemiologists, health scientists, physicians from CDC’s Tuberculosis Elimination program, and a CDC library specialist, was convened to conduct the systematic literature review using methods adapted from the Guide to Community Preventive Services (*2*,*3*). The library specialist used a systematic search strategy to identify and retrieve intervention studies on the use of 3HP to treat LTBI that were published from January 2006 through June 2017 and indexed in the MEDLINE, Embase, CINAHL, Cochrane Library, Scopus, and Clinicaltrials.gov databases. To identify missed studies, reference lists from included articles were reviewed, and CDC’s TB experts were consulted. This review included English language articles that met the following criteria: 1) the study design was randomized controlled trial, quasi-experimental, observational cohort, or other design with a concurrent comparison group; 2) the target population included, but was not restricted to, persons aged ≥12 years, children aged 2–11 years, or persons with HIV infection; and 3) outcomes reported were prevention of TB disease, treatment completion, adverse events while on 3HP, discontinuation as a result of adverse events while on 3HP, or death while on 3HP.

Two reviewers from the CDC Work Group independently screened citations obtained from the search and retrieved full-text articles in the relevant literature to be synthesized. Using a standard data abstraction form, the reviewers abstracted data on intervention characteristics, outcomes of interest, demographics, benefits, harms, considerations for implementation, and evidence gaps. Each study was also assessed for threats to internal and external validity per Guide to Community Preventive Services standards (*2*,*3*). Any disagreement between reviewers was resolved by consensus of the CDC Work Group members.

The CDC Work Group reviewed 292 citations retrieved from the librarian’s search. Of these, 30 full-text articles were ordered and screened for inclusion. No eligible studies including children aged <2 years were identified. In total, 19 articles representing 15 unique studies were included in the meta-analysis. Findings from the meta-analysis indicated that 3HP is as safe and effective as other recommended LTBI regimens and achieves significantly higher treatment completion rates. Complete results of the systematic review and meta-analysis have been published elsewhere (*4*). Overall, the majority of included studies were of greatest design suitability and good quality of execution, as defined by the Guide to Community Preventive Services (*2*,*3*). Issues related to poor reporting of appropriate analytic methods and possible selection bias were the most common limitations assigned to the body of evidence.

Recently published randomized control trials that were heavily weighted in the meta-analyses and drug interaction studies (*5*–*9*) are summarized as follows:

**Study of 3HP in children.** A large randomized clinical trial of 3HP administered by DOT, which included children aged 2–17 years, demonstrated that 3HP was as well-tolerated and as effective as 9 months of daily isoniazid (9H) for preventing TB (*5*). The trial also reported that 3HP was safe and had higher treatment completion rates than 9H (*5*). Data on the safety and pharmacokinetics of rifapentine in children aged <2 years are not available.

**Studies of 3HP in persons with HIV infection, including AIDS.** In 2011, CDC recommended the 3HP regimen for treatment of LTBI in persons with HIV infection, including AIDS, who are otherwise healthy and who are not taking antiretroviral medications (*1*). Since that time, additional data confirm not only the effectiveness of 3HP in persons with HIV infection who are not taking antiretroviral therapy, but also demonstrate the absence of clinically significant drug interactions between once-weekly rifapentine and either efavirenz or raltegravir in persons with HIV infection who are treated with those antiretroviral medications (*4*,*6*–*8*).

**Study of self-administered therapy.** A randomized clinical trial demonstrating noninferior treatment completion and safety of 3HP-SAT compared with 3HP-DOT in persons aged ≥18 years in the United States provides the primary evidence on 3HP administration by SAT (*9*). The 3HP-SAT regimen has not been studied in randomized controlled trials in persons aged <18 years.

## Expert Consultation

In July 2017, CDC met with nine non-CDC subject matter experts in TB and LTBI diagnosis, treatment, prevention, surveillance, epidemiology, clinical research, pulmonology, pediatrics, HIV/AIDS, public health programs, and patient advocacy. CDC presented the systematic review results and proposed recommendations to the experts, who provided 1) individual perspectives on the review; 2) experience with implementation of the 3HP regimen in various settings and populations; and 3) individual viewpoints on the proposed updates. Subject matter experts from programs prescribing 3HP described benefits of this regimen, including increased acceptance and completion of treatment. Some experts reported that several health departments are currently using 3HP, with high treatment completion, in children as young as age 2 years. Some noted that the 2011 recommendation to administer 3HP by DOT limits use of the regimen. In December 2017, CDC solicited input from ACET and members of the public for incorporation into the final recommendations.

With regard to pediatric use, the 2011 recommendations had included limited use of the 3HP regimen for treatment of LTBI in children aged <12 years (*1*). New data on efficacy and safety of 3HP in children were determined sufficient to recommend the 3HP regimen for treatment of LTBI in children aged ≥2 years (*4*).

Concerning patients with HIV infection, information about interactions between specific antimycobacterial agents, including rifamycins (e.g., rifampin, rifabutin, and rifapentine) and antiretroviral agents, is available in the U.S. Department of Health and Human Services Guidelines for the Use of Antiretroviral Agents in HIV-1-Infected Adults and Adolescents. These frequently updated guidelines include a section addressing management of LTBI in persons with HIV coinfection and tables with information on drug interactions.* Use of concomitant LTBI treatment and antiretroviral agents should be guided by clinicians experienced in the management of both conditions.

In 2011, CDC recommended use of the 3HP regimen by DOT (*1*). Treatment completion rates are highest when the regimen is administered by DOT (*4*). However, the burden and expense of DOT is greater than that for SAT (*9*). During the expert consultation and again during review by ACET, some subject matter experts strongly recommended permitting use of SAT, when combined with clinical monitoring, in children aged ≥2 years. Based on this expert opinion, ACET formally recommended expansion of the option of parentally administered SAT to children. Some experts still prefer DOT for treating LTBI in children aged 2–5 years, in whom risk for TB progression and severe disease is higher than that in older children and adults. Health care providers should make joint decisions about SAT with each individual patient (and parent or legal guardian), considering program resources and the patient’s age, medical history, social circumstances, and risk factors for progression to severe TB disease. Subject matter experts stressed the importance of educating providers and patients about 3HP.

## Recommendations

Based on evidence on effectiveness, safety, and treatment completion rates from the systematic review, and after consideration of viewpoints from TB subject matter experts and input from ACET and the public, CDC continues to recommend 3HP for treatment of LTBI in adults and now recommends use of 3HP 1) in persons with LTBI aged 2–17 years; 2) in persons with LTBI who have HIV infection, including AIDS, and are taking antiretroviral medications with acceptable drug-drug interactions with rifapentine; and 3) by DOT or SAT in persons aged ≥2 years.

The health care provider should choose the mode of administration (DOT versus SAT) based on local practice, individual patient attributes and preferences, and other considerations, including risk for progression to severe forms of TB disease. Use of concomitant LTBI treatment and antiretroviral agents should be guided by clinicians experienced in the management of both conditions (Box 1).

BOX 1 **Updated recommendations for once-weekly isoniazid-rifapentine for 12 weeks (3HP) for the treatment of latent tuberculosis infection**

CDC continues to recommend use of the short-course combination regimen of once-weekly isoniazid-rifapentine for 12 weeks (3HP) for treatment of latent tuberculosis infection (LTBI) in adults. With regard to age limits, HIV infection, and administration of the treatment, CDC now also recommends the following:

- use of 3HP in persons aged 2–17 years;
- use of 3HP in persons with LTBI who are living with human immunodeficiency virus (HIV) infection, including acquired immunodeficiency syndrome (AIDS) and taking antiretroviral medications with acceptable drug-drug interactions with rifapentine*; and
- use of 3HP by directly observed therapy (DOT) or self-administered therapy (SAT) in persons aged ≥2 years; the health care provider should choose the mode of administration (DOT versus SAT) based on local practice, individual patient attributes and preferences, and other considerations, including risk for progression to severe forms of tuberculosis disease.

* https://aidsinfo.nih.gov/guidelines/html/1/adult-and-adolescent-arv/367/overview.

**Patient monitoring and adverse events.** Hepatic enzymes and other blood tests should be performed for certain patients before initiation of 3HP therapy (Box 2). Approximately 4% of all patients using 3HP experience flu-like or other systemic drug reactions, with fever, headache, dizziness, nausea, muscle and bone pain, rash, itching, red eyes, or other symptoms (*4*,*10*). Approximately 5% of persons discontinue 3HP because of adverse events, including systemic drug reactions (*4*,*10*); these reactions typically occur after the first 3–4 doses, and begin approximately 4 hours after ingestion of medication (*10*). Hypotension and syncope have been reported rarely (two cases per 1,000 persons treated) (*4*,*10*). If symptoms suggestive of a systemic drug reaction occur, patients should stop 3HP while the cause is determined. Symptoms usually resolve without treatment within 24 hours. Neutropenia and elevation of liver enzymes occur uncommonly (*4*,*10*). CDC recommends that health care providers educate patients to report adverse events. Patient use of symptom checklists might facilitate timely recognition and reporting.^†^

BOX 2 **Guidance to health care providers during treatment of latent tuberculosis infection (LTBI) with a combination regimen of isoniazid and rifapentine in 12 once-weekly doses (3HP)**

- Evaluate all patients for active tuberculosis disease both before and during treatment of LTBI.
- Inform the patient or parents or legal guardians about possible adverse effects and instruct them to seek medical attention when symptoms of possible adverse reaction first appear; particularly drug hypersensitivity reactions, rash, hypotension, or thrombocytopenia.
- Conduct monthly evaluations to assess treatment adherence and adverse effects, with repeated patient education regarding adverse effects at each visit.
- Order baseline hepatic chemistry blood tests (at least aspartate aminotransferase [AST]) for patients with the following specific conditions: human immunodeficiency virus infection, liver disorders, postpartum period (≤3 months after delivery), regular alcohol use, injection drug use, or use of medications with known possible interactions.
- Conduct blood tests at subsequent clinical encounters for patients whose baseline testing is abnormal and for others at risk for liver disease. Discontinue 3HP if a serum AST concentration is ≥5 times the upper limit of normal in the absence of symptoms or ≥3 times the upper limit of normal in the presence of symptoms.
- In case of a possible severe adverse reaction, discontinue 3HP and provide supportive medical care. Conservative management and continuation of 3HP under observation can be considered in the presence of mild to moderate adverse events as determined by health care provider.

Rifapentine is a rifamycin compound; like rifampin, it induces metabolism of many medications. CDC recommends monitoring of patients when 3HP is prescribed with interacting medications (e.g., methadone or warfarin). Rifapentine can reduce the effectiveness of hormonal contraceptives; therefore, women who use hormonal birth control should be advised to add, or switch to, a barrier method. Women should be advised to inform their health care provider if they decide to try to become pregnant or become pregnant during 3HP treatment.

Because altered dosing might reduce effectiveness or safety, patients on 3HP SAT should be encouraged to record medication intake and report deviations from the prescribed regimen. Persons on 3HP regimens should be evaluated monthly (in person or by telephone) to assess adherence and adverse effects.

Additional studies are needed to understand the pharmacokinetics, safety, and tolerance of 3HP in children aged <2 years; adherence and safety of 3HP-SAT in persons aged <18 years; and safety of 3HP during pregnancy (*4*).

Any LTBI treatment–associated adverse effect leading to hospital admission or death should be reported by health care providers to local or state health departments for inclusion in the National Surveillance for Severe Adverse Events Associated with Treatment for LTBI (e-mail: ltbidrugevents@cdc.gov). Serious drug side effects, product quality problems, and therapeutic failures should be reported to the Food and Drug Administration’s MedWatch program (https://www.fda.gov/Safety/MedWatch/HowToReport/default.htm) or by telephoning 1-800-FDA-1088.

Additional information regarding 3HP is available at https://www.cdc.gov/tb/publications/ltbi/ltbiresources.htm. Questions also can be directed to CDC’s Division of Tuberculosis Elimination by e-mail (cdcinfo@cdc.gov) or by telephoning 800-CDC-INFO (800-232-4636).

## Footnotes

* https://aidsinfo.nih.gov/guidelines/html/1/adult-and-adolescent-arv/367/overview.

† Examples of patient’s medication intake log and symptoms checklists are available at https://www.cdc.gov/tb/publications/pamphlets/12-doseregimen.htm.
